# Society Notes

**Published:** 1888-09

**Authors:** 


Yours truly,

H. A. West.

### Society Notes.

Travis County Medical Society.—At the last meeting of this flourishing Society, held in Austin, September 6th (inst.,) Dr. John Preston, first assistant physician, State Lunatic Asylum, was elected President, Dr. Justus Duffau Vice-President, and Dr. B. F. Church Secretary and Treasurer.

Limestone County Medical Society.—We learn from Dr. Lewis, of Mexia, that the physicians of Limestone county organized with a membership of twenty-five. They are all veterans and workers—all active practitioners—and the doctor says "there are no drones." Dr. Lewis was elected President. We hope to have a report of their work. Dr. Lewis will contribute to the Journal occasionally.

Austin District Medical Society.—The fourth quarterly meeting of the Austin District Medical Society will be held in Austin, on the 20th inst. A large attendance is expected, it being the occasion for the first annual election of officers. There will be a session,—morning, afrernoon, and evening; and after the latter, the occasion will be celebrated by a banquet or supper. A committee from the Travis County Medical Society has been appointed to co-operate with a committee from the District Society, to prepare for the occasion.

THE CHEROKEE COUNTY MEDICAL SOCIETY met recently (Aug. 20), in Alto. There were a large number of physicians present, most of whom were present at the organization. Ten new members were admitted.

Reports were made by the Committee on Credentials, Drs. Spring, Jameson and Guinn, and Correspondence, Drs. Evans and Frazer, and also by the committee to whom was referred a resolution to change the manner of voting from ballot to *viva voce*, committee, Drs. Collins, Frazer and Jamison. Drs. W. G. Jamison, N. W. Spring and J. H. Evans, the essayists for the occasion, each read a paper, the subjects being "Vomiting in Pregnancy," "Eczema," and "Forceps Delivery." Papers discussed by Dr. A. H. McCord, and others. A resolution was adopted taxing each member fifty cents, to pay for the publication of papers read at the meetings of the Society. [This JOURNAL will be pleased to publish such papers, free of charge, if found worthy of being presented to the profession.—ED.]

By resolution of Dr. Frazer, the physicians of Cherokee county were once more cordially invited to meet with the Society, at the Jacksonville meeting, in October.

Drs. A. H. McCord and J. N. B. Guinn each read a paper. [Reporter does not state what disposition was made of them, nor report any discussion which followed. We beg to remind him that without a report of discussion, the mere details of business is dry reading. Hope he will be more explicit at next report.—ED.]

Drs. B. F. Brittain and Collins, of Jacksonville; Barton and Wilson, of Alto, and Guinn, of Rusk, were appointed essayists for next meeting.

J. F. JOHNSON, Secretary.

---

A SAMPLE LETTER TO THE JOURNAL.—"The JOURNAL is always a welcome visitor. It is bright, interesting and instructive. You may justly be proud of it. We wish you increased success and profit in its publication the ensuing year. With best wishes,

Yours truly,

